# Droughts in India from 1981 to 2013 and Implications to Wheat Production

**DOI:** 10.1038/srep44552

**Published:** 2017-03-15

**Authors:** Xiang Zhang, Renee Obringer, Chehan Wei, Nengcheng Chen, Dev Niyogi

**Affiliations:** 1 State Key Laboratory of Information Engineering in Surveying, Mapping, and Remote Sensing (LIESMARS), Wuhan University, Wuhan 430079, China; 2 Department of Agronomy-Crops, Soil, Environmental Science, Purdue University, West Lafayette, IN 47906, USA; 3 Department of Earth, Atmospheric, and Planetary Sciences, Purdue University, West Lafayette, IN 47906, USA; 4 Lyles School of Civil Engineering, Purdue University, West Lafayette, IN 47906, USA; 5 Collaborative Innovation Center of Geospatial Technology, Wuhan 430079, China

## Abstract

Understanding drought from multiple perspectives is critical due to its complex interactions with crop production, especially in India. However, most studies only provide singular view of drought and lack the integration with specific crop phenology. In this study, four time series of monthly meteorological, hydrological, soil moisture, and vegetation droughts from 1981 to 2013 were reconstructed for the first time. The wheat growth season (from October to April) was particularly analyzed. In this study, not only the most severe and widespread droughts were identified, but their spatial-temporal distributions were also analyzed alone and concurrently. The relationship and evolutionary process among these four types of droughts were also quantified. The role that the Green Revolution played in drought evolution was also studied. Additionally, the trends of drought duration, frequency, extent, and severity were obtained. Finally, the relationship between crop yield anomalies and all four kinds of drought during the wheat growing season was established. These results provide the knowledge of the most influential drought type, conjunction, spatial-temporal distributions and variations for wheat production in India. This study demonstrates a novel approach to study drought from multiple views and integrate it with crop growth, thus providing valuable guidance for local drought mitigation.

As an extreme event, drought severely affects global plant growth and food production^1^,^2^,^3^. Considering climate change and anthropogenic influences^4^, an overall enhanced drought risk for crop yield in the future is well documented^5^,^6^,^7^. In India, this risk is greater due to deviated monsoon rains^8^,^9^,^10^, depleted groundwater^11^, and the pressure of food demand from a population of 1.252 billion^12^,^13^.

Drought in India has been studied since the 1960 s^14^,^15^. With regard to the drought mechanism, it was found that prolonged ‘breaks’ in the southwest monsoon resulted in severe summer droughts in the Indian subcontinent due to upper tropospheric blocking ridges over East Asia^16^,^17^. With respect to drought monitoring, remotely sensed and *in-situ* data (e.g., precipitation, runoff, temperature, and vegetation data) have been used to assess drought condition alone^18^,^19^ or in a combination approach^20^,^21^,^22^,^23^. In addition, reanalysis products, near real-time drought monitoring, and new drought indices have also been studied in India^24^,^25^,^26^. From the aspect of drought distribution and trend, several studies have found distinctive drought frequencies existed in different regions of India^27^,^28^,^29^,^30^. Besides that, Ojha *et al*.^31^ predicted that drought events were expected to increase in the west central, peninsular, and central northeast regions of India in 2050–2099. Considering drought impact, Subash and Mohan^32^ found that the monthly distribution of monsoon rainfall in terms of Standardized Precipitation Index (SPI) accounted for a 44% yield variability in rice. Similarly, SPI-7 in April and May was found to be substantially correlated with wheat production^33^.

One of our recent studies also analyzed drought trends and variability in India for the period 1901–2004^34^. Results indicated an increasing trend in drought severity and frequency. More regional droughts in the agriculturally important southern coast India, central Maharashtra, and Indo-Gangetic plains were also highlighted indicating higher food security and socioeconomic vulnerability. However, this preliminary study only focused on precipitation-based meteorological drought. In addition, it was recognized that while drought stress could be identified, the implications on crop production required a more comprehensive consideration of crop phenology.

Building off these studies, it is now possible to reconstruct major types of drought by using long-term multi-sensory datasets, including meteorological, hydrological, soil moisture, and vegetation droughts. Definitions of drought types can be found in Dracup *et al*.^35^ and Wilhite and Glantz^36^, while in this study, we separate the conventional term of agricultural drought into two types of drought (i.e., soil moisture, and vegetation droughts). This new approach will help us to have a refined view on drought transformations. Therefore, it is timely to conduct a comprehensive analysis of the distribution, duration, severity, and trends of these droughts simultaneously as their interactions are still relatively unknown, especially in India. The relationship between these four different kinds of drought and crop production is also lacking in the previous studies^32^,^33^,^34^. For example, questions such as which type of drought has the most significant impact on wheat yield loss, and does this relationship vary with time, need to be addressed. Furthermore, as India has benefited from extensive irrigation, conventional indices such as soil moisture index will not be able to fully depict the water-stress condition. Here a comprehensive approach is presented to investigate multiple droughts in India so as to assess their influences on wheat production.

For the first time, four kinds of drought, including meteorological, hydrological, soil moisture, and vegetation were studied at the same time using occurrence, spatial-temporal evolution, severity, duration, and evolution from 1981 to 2013. Particular attention was offered to the drought evolution during the wheat growing (spanning from October through April). The specific goal of this study is to improve the understanding of different droughts and their influences on wheat yield from a finer and systemic view thereby increasing the efficiency of linking drought stress with impact on crop yield at a regional scale.

## Results and Discussion

### Analysis of retrospective droughts from 1981–2013

Historical droughts were reconstructed using gridded observed precipitation, model-simulated total runoff, soil moisture, and remotely-sensed vegetation data. A time sequence of mean SPI, Standardized Runoff Index (SRI), Standardized Soil Moisture Index (SSI), and Vegetation Condition Index (VCI) in the study area for every month from 1981–2013 can be found in Supplementary Fig. S1. It is not easy to determine whether the study area became drier or wetter by visual inspection alone. While the persistence of soil moisture and hydrological conditions is distinctive as these two exhibit less variability relative to precipitation. When precipitation anomalies occurred, corresponding changes in hydrology and soil moisture were often observed immediately. However, to quantify the above preliminary judgements and gain more precise knowledge, more quantitative analyses were conducted.

The occurrence of droughts for different years was listed first (details in Supplementary Tables S1–4). Years when at least three kinds of droughts occurred during the wheat growth season are 1985, 1990, 1993, 1997, 1999, 2000, 2001, 2004, 2006, and 2010. They were judged as significantly drought-impacted years for wheat production. 60% of years when meteorological droughts occurred were after 2000, with 91% of them concentrated in January to February while 43% of hydrological droughts occurred in the 1990 s, and 53% of vegetation droughts occurred before 1993. In addition, vegetation drought was more than double in February than in other months. It was also found that the severity of most meteorological and soil moisture droughts was equivalent of D1 (as used in the United States or global drought monitor), while the other two reaches D3 or even D4 (Severity thresholds are shown in Supplementary Table S8). For this study area, hydrological and vegetation droughts are more influential based on their level of severity, and supportive evidence of the areal extent is also provided.

The top drought year by spatial extent or severity are listed for all wheat growth months and all four drought types (shown in Tables 1 and 2). It is found that 19 out of 28 years with the largest spatial extent are well correlated to years with the most severe drought conditions. It is interesting to note that in October 2000, the largest area of meteorological, hydrological, and vegetation drought occurred at the same time as the most severe hydrological and vegetation drought. Corresponding to the two water-stress sensitive stages for crops (Heading and Anthesis), the most influential droughts occurred in 1985 and 2006 when at least four of the top droughts occurred with the maximum areal extent or highest severity (wheat phenology information is shown in Supplementary Table S10). In terms of severity, hydrological and vegetation droughts are usually more severe than meteorological and soil moisture droughts. This difference is also valid in terms of spatial extent. It is also notable that even the most severe meteorological droughts in study area are mainly featured by local and moderate impact, with averaged 44.8% of the whole spatial extent and D1 severity. In addition, severe meteorological droughts only occurred in January 2007, February 2006, and March 2004.

**Figure 1:**
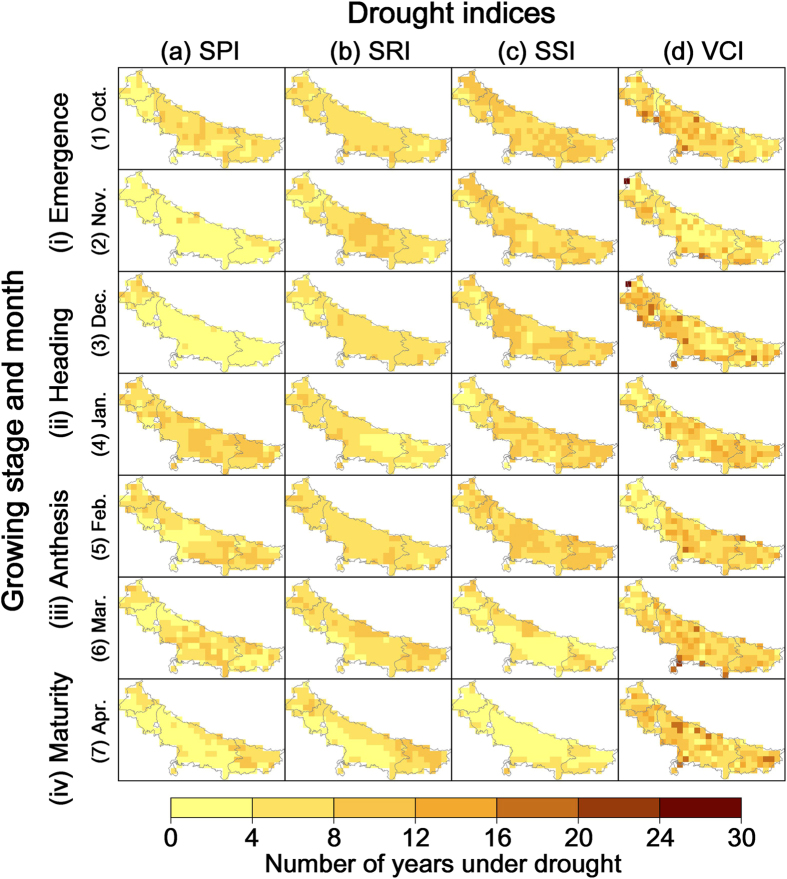
Temporal extent of droughts for every month of the crop growing season. The temporal extent data was calculated by Matlab R2014b (Version 8.4, URL: http://www.mathworks.com) [Software] with the method described in the next section. Then the data was input into ArcGIS Desktop (Version 10.2.3348, URL: http://www.esri.com) [Software] to generate this color rendered map layer. Administrative boundary layer of the study area was obtained from DIVA-GIS (URL: http://www.diva-gis.org/Data). DIVA-GIS provides free spatial data for geographical information system. Finally all these maps were organized and labeled in the Microsoft Visio Professional 2013 (Version 15.0.4569.1506, URL: https://products.office.com/en-us/visio) [Software].

At pixel level (grid space is 0.5 degree), the number of years with meteorological, hydrological, soil moisture, and vegetation drought (severity of D1 or higher) was analyzed. As shown in Fig. 1, the temporal extent of droughts provides the number of years under drought conditions for every month of the crop growth season. It was found that almost all study areas experienced more than 8 times the average number of droughts during January for the 33 year period, while occurrences of meteorological drought in November and December were less than 4. Regarding hydrological drought, there was little monthly variation showing only about 4–8 drought years. Soil moisture drought occurred more frequently (over 8 times) from October to February, while March and April had only about 4. The temporal extent of vegetation drought is notable due to the heterogeneous distribution of occurrences in contrast to the other three. There is no significant difference for different months, however, in some months, the occurrence varied greatly at different pixels (i.e., from under 4 to above 16). No obvious spatially concentrated region was found.

This result suggested that during the entire wheat growth season, there was no large monthly difference for hydrological and vegetation droughts. Meteorological droughts are particularly concentrated in January, and soil moisture droughts during October to February. The different number of years in which a grid cell was under drought conditions was spatially highlighted across the study domain, especially for vegetation drought. Overall, October, January, and February are judged as three drought-prone months when all four kinds of drought usually occur at the same time.

### Concurrent droughts

In addition to the above retrospective analysis of different drought types, concurrent drought analysis was also conducted. The concurrent drought during the wheat growing season was analyzed from four aspects, including temporal distribution (year and month), spatial distribution, concurrent types, and frequency. The conjunctional feature of regional droughts in each month during the wheat growing season is provided from a regional perspective in Supplementary Table S5. Firstly, it was found that 17 concurrent droughts occurred in 12 years during the wheat growth in 1981–2013. February was identified as a multi-drought prone month, with over 41% of the historical concurrent droughts. There were no concurrent droughts in November. Besides that, two-drought based conjunctions account for over 76% of concurrent types, and only February 1985 was impacted by all four kinds of droughts. The wheat production experienced the highest frequency of concurrent drought in 1993, with three times of vegetation drought in October, December, and February, respectively. Additionally, it was found that over 88% concurrent droughts included the hydrological drought. This result not only showed the high frequency of hydrological drought, but also suggested the important and interconnected role of not just precipitation but more so surface hydrology in the study area.

**Figure 2:**
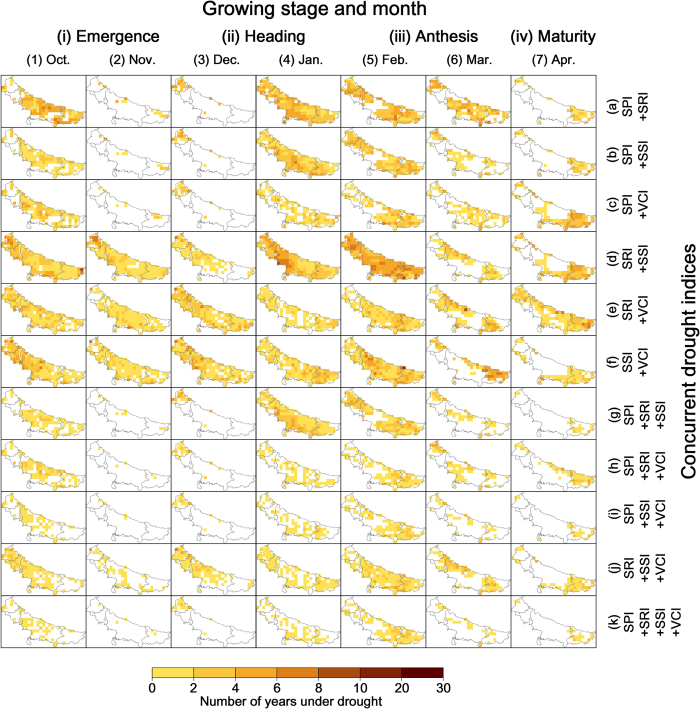
Temporal extent of concurrent droughts for every month of the crop growing season. The temporal extent data was calculated by Matlab R2014b (Version 8.4, URL: http://www.mathworks.com) [Software] with the method described in the next section. Then the data was input into ArcGIS Desktop (Version 10.2.3348, URL: http://www.esri.com) [Software] to generate this color rendered map layer. Administrative boundary layer of the study area was obtained from DIVA-GIS (URL: http://www.diva-gis.org/Data). DIVA-GIS provides free spatial data for geographical information system. Finally all these maps were organized and labeled in the Microsoft Visio Professional 2013 (Version 15.0.4569.1506, URL: https://products.office.com/en-us/visio) [Software].

To understand the spatial distribution of concurrent droughts in all eleven kinds of conjunctions, the number of years under concurrent droughts in each grid was illustrated in Fig. 2. Overall, the most number of years under concurrent drought ranges from one to eight. During wheat growing season, October, January, and February were found to have the most concurrent droughts regardless of the spatial location. November and December have three common combinations, including hydrological with soil moisture drought, hydrological with vegetation drought, and soil moisture with vegetation drought. These concurrent types are also the most common types when comparing with others. Besides that, it was found that no matter what kind of the concurrent drought occurred in April, most of them were usually distributed in the southern part of the domain. The conjunction of all four kinds of drought mostly occurred in October, January, and February.

### Drought evolution

**Figure 3:**
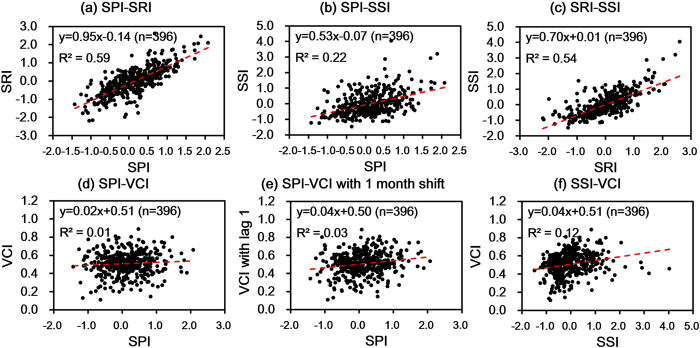
Relationship between domain-mean monthly (**a**) SPI-SRI, (**b**) SPI-SSI, (**c**) SRI-SSI, (**d**) SPI-VCI, (**e**) SPI-VCI with a 1 month shift, and (**f**) SSI-VCI from 1981–2013. Linear regression equation with sample size (n) and coefficient of determination (R^2^) were shown.

Based on monthly drought data, the evolution process is shown in Table 3 and Fig. 3. It was interesting to find that there was no time lag between these four kinds of drought, except for the evolution from meteorological to vegetation drought. This result indicates that in the study area, the transformation between meteorological, hydrological, and soil moisture drought is typically within 1 month for the wheat belt. It also appears that it takes less than 1 month for soil moisture drought to become vegetation drought, but the complete drought evolution (from meteorological to vegetation drought) lasts about 1 month. This is to say, with a sharp decrease in rainfall, there can be a rapid evolution to meteorological, hydrological, and soil moisture droughts in the same month. Vegetation will show significant water stress only after this month. This suggests that for the future assessments, at least weekly drought data is needed in order to have a more detailed view of evolution.

The reason for this rapid evolution of droughts in the study region is an interesting study question. There are several factors that can only be conjectured within the scope of the present study. The study region is already known as a global hotspot for land–atmosphere coupling in global climate model studies. Preliminary review of a short span of satellite and reanalyses data suggests that for drought to trigger the rainfall deficit occurs first, then this especially in crop growing region appears to a rapid evapotranspiration (ET) increase possibly as a vegetation growth and temperature feedback induced by rainfall anomaly. This then creates a larger effective precipitation deficit, which reflects in the soil moisture loss. These processes are typically a week-long time scale (but confounded within the monthly time scale analyzed in this study). The hydrological response appears to be a separate reduction and while the soil moisture and ET play a role they are likely more synergistic than causal. This conjecture and initial analysis will be assessed in a more detailed follow up study using higher temporal resolution soil moisture fields, coupled regional meteorological studies assessing the local moisture recycling potential, dynamic vegetation growth and reanalyses fields. The reduction in rainfall and the high demand of water from crop on the ground linked with the intensive water usage accentuates the drought evolutions. In reality this seems to be offset by the irrigation in this region (which is analyzed next).

Results from the linear regression analysis suggested 59% of hydrological variations can be explained by precipitation anomalies, 54% of soil moisture by hydrology, while the coefficient of determination between precipitation and soil moisture is only 0.22. This result indicates that runoff is primarily controlled by rainfall, and is the main water source for soil in this study area. Interestingly however, there are 31 months with hydrological drought but without soil moisture drought, but only 18 months with both kinds of drought. The former is about 72% more than the latter. Considering natural con-occurrence of the processes that cause these two droughts in the same month, their numbers were expected to be comparative. This result demonstrated the signature of ground water-based irrigation agriculture in the study area (after the Green Revolution in India in the 1960 s to 1970 s). The Green Revolution improved wheat production significantly in India by adopting high-yield variety seeds, chemical fertilizers, pesticides, and irrigation^37^. Our results are thus indicative of the irrigation activity where in more surface water had been pumped out for irrigation when dealing with the drought stress. Both soil moisture and precipitation have low correlations with simultaneous or 1 month shifted vegetation conditions. This result further revealed the considerable anthropogenic and other factor impacting crop management in this study area. Given these types of drought evolution characteristics, it can be suggested that rapid mitigation strategies would be required after meteorological drought occurrence in this region.

### Drought trends

Results of drought trend analysis, including the statistical mean value of duration, frequency, areal extent with linear regression, Mann-Kendall analysis, and latitudinal variation are provided below (see more details in Supplementary Tables S6–8 and Fig. S2).

From the perspective of duration trends (see Supplementary Table S6), it was found that only meteorological drought lingered slightly longer since 1981, from 1 to 1.2 months per drought event. At the same time, the duration of the other three droughts shortened to 1.1, 1.4, and 1.3 months per event and the mean duration of soil moisture drought fell sharply by half. This result demonstrated the mean duration of all drought types was slightly longer than 1 month in the study area. In other words, more “flash droughts” occurred, compared with multi-months or years-long drought.

From the view of frequency (see Supplementary Table S7), which means how many times drought events occurred in every decade, meteorological and soil moisture drought exhibited an upward trend (from 2 to 11, and from 5 to 10), while the other two decreased (from 13 to 11, and from 20 to 9). That is to say, there are increased rainfall and soil moisture anomalies with fewer anomalies of runoff and vegetation. In the latest decade, there was an average of 10 times (with a standard deviation of 0.96) of every type of droughts.

Regarding the areal extent (see Supplementary Table S8 and Fig. S3), overall, only meteorological drought impacted increasingly larger areas, reaching 18.0% of the whole study area in the 2000 s from 12.7% in the 1980 s. In recent decades, the other three droughts are more likely to form as local drought events. The areal extent of every hydrological and soil moisture drought decreased slightly from 21.4% to 18.9%, and from 24.3% to 19.9%, respectively while the area of vegetation drought shrank from 32.9% to 23.2%. The mean areal extent for all four drought types is 20% in the latest decade with a standard deviation of 2.27.

Based on this statistical analysis, more prolonged, frequent, and larger area of meteorological drought was found, which is consistent with our previous study^34^. On the contrary, hydrological and vegetation droughts were relieved by shorter duration, less frequency, and smaller areal extent. Soil moisture drought occurred more frequently, but in a local and short-term manner.

**Figure 4:**
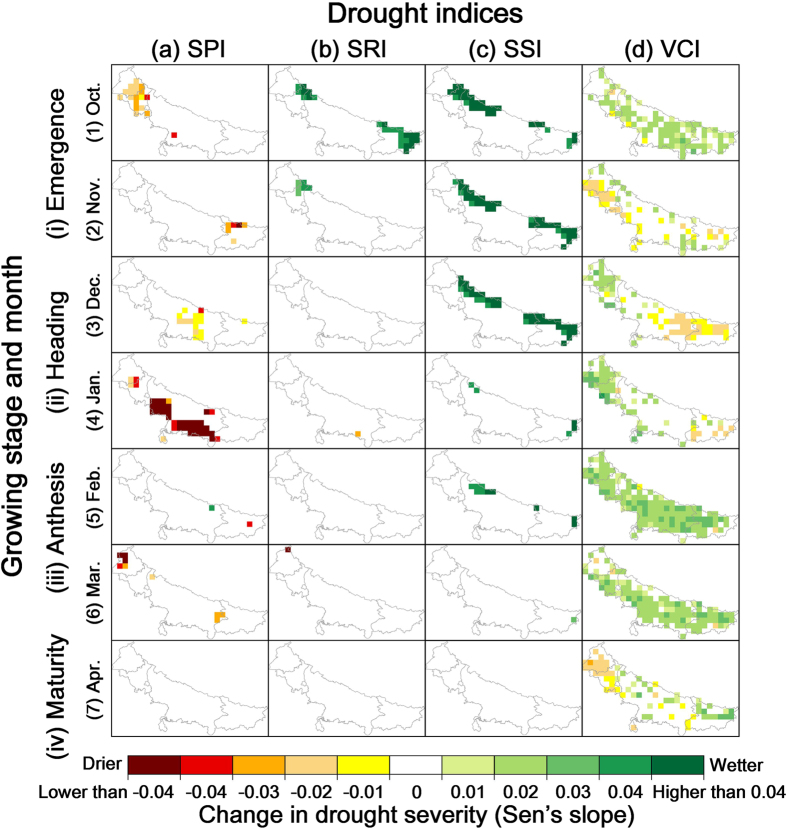
Severity trend of (**a**) meteorological, (**b**) hydrological, (**c**) soil moisture, and (**d**) vegetation drought for every month of wheat growth between 1981 and 2013. Significance level of 0.05 was applied in the Mann-Kendall analysis. The severity trend data was calculated by Matlab R2014b (Version 8.4, URL: http://www.mathworks.com) [Software] with the method described in the next section, which was realized by Jeff Burkey (URL: https://www.mathworks.com/matlabcentral/fileexchange/11190-mann-kendall-tau-b-with-sen-s-method--enhanced). Then the data was ingested into ArcGIS Desktop (Version 10.2.3348, URL: http://www.esri.com) [Software] to generate this color rendered map layer. Administrative boundary layer of the study area was obtained from DIVA-GIS (URL: http://www.diva-gis.org/Data). DIVA-GIS provides free spatial data for geographical information system. Finally all these maps were organized and labeled in the Microsoft Visio Professional 2013 (Version 15.0.4569.1506, URL: https://products.office.com/en-us/visio) [Software].

For the spatial domain of monthly drought severity trends, the Mann-Kendall analysis results are shown in Fig. 4. It is easy to find distinct contrasts between trends in different drought types during the wheat growth season. Meteorological drought generally became more serious in the northeastern areas in October and March, and in December and January for the central southern regions. In particular, the magnitude of change in January is remarkable, suggesting a much greater rainfall deficit. No significant trend was found in February and April. For hydrological and soil moisture droughts, part of the region near the upper boundary was notably relieved especially in October, November, and December, while other areas and other months did not show a significant severity trend. Due to the high sensitivity of soil moisture stress from December to March for wheat yield, this result suggested the soil water supply became more favorable in these sub-regions likely due to irrigation. Vegetation drought trends are significant due to the larger areal extent. Overall, vegetation became much drier in the northeast areas in November and April, and in December for the south, while other regions and other months became wetter or showed little trend. Therefore, in the last three decades, only meteorological and vegetation droughts increased in severity for certain sub-regions. This result highlighted different susceptible regions for each month to response more serious meteorological and vegetation droughts.

To determine detailed latitudinal drought trends, change in the number of years under drought by latitudinal value since 1981 is shown in Supplementary Fig. S3. Varied change patterns were found for meteorological drought, but overall, there was some tendency for a southern shift in January, while for other months, they are spatially concentrated in the north or south. No latitudinal movement was found in hydrological drought. Regions above 28°N experienced more serious soil moisture drought, and is obvious from October to December. Vegetation drought is spatially concentrated above 28°N in October to December, while below 28°N in February to March. Understanding the reason for this spatial discrimination is another notable feature that needs to be studied in a follow up study.

### Relationship of drought with crop yield

The above analysis demonstrated the relationship between drought and wheat growth from the perspective of occurrence, distribution, and trend. The numerical relationship between them is also presented and shown in Table 4 and Supplementary Fig. S4. Interestingly, it was found that during the entire wheat growth season, only soil moisture and vegetation drought correlated well with final wheat yields for certain months. Generally speaking, the soil moisture index in the Emergence stage (October and November) is significantly related to wheat yield anomaly (correlation coefficient r was 0.38 and 0.45, with the p value of 0.03 and 0.01). The vegetation condition index is much more closely correlated in the Anthesis stage (February and March), as the correlation coefficient r was 0.75 and 0.74, with both p values of 0.00. In addition, soil moisture and vegetation drought indices both have high correlation coefficients in October and February. No significant correlation was found in the Heading and Maturity stages (i.e., December, January, and April). The reason that correlation coefficients between vegetation drought and yield loss are low in April is probably related to the impact from harvest activity. Overall, these results demonstrated that VCI in the Anthesis stage was a good indicator for final yield loss. Alternative indicators are VCI in October and SSI in November. SPI is the default drought index used worldwide, especially in developing countries, and results indicate that it should be used with caution for agricultural drought assessment. These results also highlight the need to address drought stress and food security discussions for climate studies in a more comprehensive manner with explicit consideration of crop phenology and evolution of different drought types. Further, future studies and assessments should exercise caution in correlating rainfall deficits or SPI-like estimates for current and future climate to crop yield loss or food security. Consideration of the role of crop phenology, drought evolution, and local management practices is necessary in developing drought impact assessments in a more systems approach.

## Data and Methods

### Site description

Next to rice, wheat is the most important food-grain of India and is the staple food of millions in that region. The Indo-Gangetic Plain (IGP) region of India has been referred to as the ‘bread basket’ or ‘food bowl’ of the country. Punjab, Haryana, Uttar Pradesh, and Bihar are the four prominent wheat producing states in IGP and were selected as the primary area of study (Supplementary Fig. S5). These states mainly belong to the Northwestern and Northeastern Plains Zone based on agro-climatic conditions. They account for about 58% of wheat area and about 67% of the total wheat production in India in 2013–2014, according to the Department of Agriculture, India. In fact, these areas have earned the distinction of being called the “Granary of India”.

The area of wheat growth in the study area increased slowly from about 1,400 ha to 1,800 ha, but production rose to more than 60 million tonnes from about 25 million tonnes due to yield increases (Supplementary Fig. S6). The overall yield trend was notable increasing from 45 to 85 million tons per million ha, although fluctuations were noted. From 1980–1990 and 2002–2014, the actual yield was below the trend, while from 1991–2001, the actual yield was higher than average.

Monthly mean air temperatures in the study area ranged from about 10 °C in December and January to more than 30 °C from May to August (Supplementary Fig. S5). The temporal variation of rainfall was significant: 64.85% of the annual precipitation was concentrated in the monsoon season (July to September), while the total amount of precipitation was only 25.4 cm during the wheat growth season (i.e., October to April). However, the amount of rainfall required for wheat cultivation varies between 30 cm and 100 cm. Therefore, the study area was classified as a drought-prone area for wheat production, which is also highlighted in our previous study^34^. Since rainfall is not the only factor to influence wheat yield, this study will help determine the percentage of yield loss caused by different types of drought condition in India.

It is worth noting that the irrigation rate of this study area is over 40% in 2009–2010 according to the Open Government Data (OGD) Platform India. This is brought by the Green Revolution since 1960 s in India^37^. Due to this kind of human intervention, precipitation-only or soil moisture-only based drought index will not be able to truly capture the surface drought condition. Therefore, a multi-index approach is adopted to study the drought in this area.

### Drought occurrence and severity

Four widely used drought indices were selected, including the Standardized Precipitation Index (SPI), Standardized Runoff Index (SRI), Standardized Soil moisture Index (SSI), and the Vegetation Condition Index (VCI) (see the Supplementary Text and Table S9). Monthly scales of SPI, SRI, SSI, and VCI from 1981 to 2013 were used to determine occurrence and severity of meteorological, hydrological, soil moisture, and vegetation droughts, respectively. By studying soil moisture drought and vegetation drought explicitly, we can quantify changes of specific environmental variables more directly, compared with a multi-variate integrated agricultural drought index (e.g., Vegetation Drought Response Index (VegDRI)).

To obtain the value of SPI, grid precipitation data from 1981 to 2013 was obtained from Global Precipitation Climatology Centre (GPCC) full data reanalysis version 7 products. The GPCC full data reanalysis monthly product is comprised of monthly totals on a regular grid with 0.5° spatial grid spacing. Based on 67200 stations worldwide, GPCC data was regarded with high accuracy^38^,^39^,^40^. Data input for calculating SRI and SSI came from the Modern-Era Retrospective analysis for Research and Applications, Version 2 (MERRA-2) product. MERRA-2 is the first long-term global reanalysis to assimilate space-based observations of aerosols and surface landscape and represent their interactions with other physical processes in the climate system^41^. In this study, the two-dimensional, monthly mean, and time-averaged land surface product (MERRA-2 tavgM_2d_lnd_Nx) was selected. Based on that, monthly runoff and root zone soil moisture values were obtained spanning from 1981 to 2013, with spatial resolution resampled to 0.5°*0.5° from 1/2°*2/3°.

In addition to the above data, VCI was also used to quantify the vegetation deficit^42^,^43^. VCI compares the current NDVI to the range of values observed for the same period in previous years. Unlike NDVI, VCI has the capability to separate short-term weather-related fluctuations from long-term ecological changes. Lower and higher VCI values indicate bad and good vegetation state conditions, respectively. To obtain VCI, the Global Inventory Modeling and Mapping Studies (GIMMS)-NDVI from NASA was used^44^,^45^,^46^. Details of VCI computation can be found in the Supplementary Text online. The latest version, termed the third generation NDVI data set (GIMMS NDVI3g) was selected for the period from July 1981 to December 2013, with a spatial resolution of 0.5° resampled from 1/12°. Bi-weekly GIMMS NDVI3g was also averaged to a monthly mean value to match the temporal resolution of precipitation, runoff, and soil moisture.

We acknowledge that station-based data is more direct and reliable to detect local extremes. However, due to the limited availability of station-based data and the need for having concurrent variables to assess drought evolution, the above grid datasets were adopted in this study. In addition, the suitability and reliability of the above grid datasets used in drought research are well documented^18^,^19^,^20^,^21^,^22^,^23^.

### Areal extent, temporal extent, frequency, duration, and distribution of droughts

As described above, occurrences of meteorological, hydrological, soil moisture, and vegetation droughts were determined by SPI, SRI, SSI, and VCI respectively. Concurrent meteorological, hydrological, soil moisture, and vegetation droughts in the same month were determined by considering the SPI, SRI, SSI, and VCI values together. Then, the spatial/areal extent of the drought in each month was estimated by counting the total number of grid cells that experienced a drought and dividing that by the total number of grid cells in the study domain to estimate the percentage area under drought in a given period of time. To obtain the temporal drought extent, the number of years that each grid cell experienced a drought in a given month from 1981–2013 was counted. The temporal extent of concurrent droughts adopted the similar approach, while considering the occurrences of multi-droughts at the same time. For example, to calculate the concurrent meteorological drought and hydrological drought, the number of years each grid cell experienced these two droughts for the same month from 1981–2013 was counted.

The mean duration of drought in each decade was calculated as well. First the total number of drought events in one decade was counted. This is also called the frequency of drought in one decade. Then the duration of each drought event in this decade was summed up to get the total duration time. Finally, the mean duration of each drought in this decade was obtained by dividing the total duration time by drought occurrence numbers.

To analyze the latitudinal distribution of drought, every row of SPI/SRI/SSI/VCI data in the study area was first compressed to one mean value thereby transforming the drought map for every year into a column vector. The number of years under drought in each triennium was then obtained by summing up the column vectors for each three year period. Finally, all eleven triennium drought vectors were arranged by time to investigate the latitudinal trend.

### Evolution process of drought

The evolution process is a qualitative process defined by the United States National Weather Service^47^ and the National Drought Mitigation Center^48^ as the formation process from meteorological to hydrological, then to soil moisture, and finally to vegetation drought. This multi-view process is valuable to probe into the water deficit transformation in different drought related variables. However, many current studies lack quantitative analysis about this feature. In this study, the theoretical analysis on how one kind of drought can influence the others is firstly shown in Supplementary Text and Fig. S7. The time required for transformation between drought types, called time lags, was still unknown for this study area. So the cross-correlation analysis was adopted as the second step to obtain their evolution lags. To determine the degree of relevancy between the evolution process of these four kinds of droughts, linear regression analysis was also used (Supplementary Text). In this study, the anthropogenic signature of the extensive irrigation (brought by the Green Revolution in India) was evaluated by analyzing this evolution process as well. Besides that, a copula-based analysis^49^ maybe also useful to study the relationship between different kinds of drought. Both of these assessments would be considered in our future study.

### Drought trend by Mann-Kendall analysis

There are at least three different conclusions regarding drought trend: increase, decrease, and no change (see Dai^50^, Sheffield *et al*.^51^, and Mallya *et al*.^34^). In this study, a more comprehensive analysis was conducted to answer this question specifically for India’s wheat belt. Besides the above statistical analyses about areal extent, frequency, and duration changes to estimate drought trends, we also used Mann-Kendall’s trend test at a significance level of 0.05 (Supplementary Text). The Mann-Kendall test^52^,^53^ has been used in many previous studies for the detection of trends in hydrologic and climatic data^54^,^55^,^56^. In addition, Sen’s slope method^57^ was also used to quantify the magnitude of trend (Supplementary Text).

### Relationship of drought with crop yield

Wheat is mainly a winter season (“Rabi”) crop in India and is usually planted in October and harvested in April. To evaluate the relationship between drought and wheat yield, the entire growing season was selected for this study. The phenology stages of wheat were listed as emergence, heading, anthesis, and maturity (Supplementary Table S10). The sensitivity of wheat yield to soil moisture stress varied during different phenological stages (Supplementary Table S10). Therefore, the relationship between drought and wheat yield for every month during the growing season was necessary for this study. This approach is different from a general drought study, such as our antecedent study^34^. The analysis of drought impacts on crop productivity was completed by using the drought indices from October to April for every year. Besides that, it is also noted that the potential of the crop to extract water from depths varies during different stages of the crop growth^58^. Therefore, considering the soil moisture in different depths will provide a finer approach to analyze the relationship between crop and soil moisture, such as the study by Narasimhan and Srinivasan^58^. To utilize that approach, gridded soil moisture is being developed as part of a regional reanalyses and will be included in our future work.

Crop yield data was available for wheat from 1980–2014 through the Directorate of Economics and Statistics, Department of Agriculture, India (http://eands.dacnet.nic.in/) and Agricultural Statistics at a Glance 2014. These data are available by state for India. The yield anomalies index for every year was calculated as yield loss (Supplementary Text). Spearman correlation coefficients were then calculated calculated to identify relationships between the crop yield anomaly and drought indices.

## Additional Information

**How to cite this article:** Zhang, X. *et al*. Droughts in India from 1981 to 2013 and Implications to Wheat Production. *Sci. Rep.* **7**, 44552; doi: 10.1038/srep44552 (2017).

**Publisher's note:** Springer Nature remains neutral with regard to jurisdictional claims in published maps and institutional affiliations.

## Supplementary Material

Supplementary Information

## Figures and Tables

**Table 1:** Top drought year based on the spatial extent (%) estimated using domain mean drought indices for every month in the crop periods from 1981–2013.

Emergence					Heading				Anthesis			Maturity	
Year	Oct.	Year	Nov.	Year	Dec.	Year	Jan.	Year	Feb.	Year	Mar.	Year	Apr.
Meteorological Drought													
**2000**	53.5	**1996**	4.4	**1998**	11.3	**2007**	86.2	**2006**	71.1	**2004**	56.0	**1989**	30.8
Hydrological Drought													
**2000**	97.5	**1983**	98.7	**1998**	100	**2007**	95.0	**2006**	98.7	**1994**	95.6	**1999**	95.0
Soil Moisture Drought													
**1989**	87.4	**1989**	85.5	**1989**	74.8	**1990**	76.7	**2001**	99.4	**1985**	56.6	**1999**	49.1
Vegetation Drought													
**2000**	81.8	**2011**	79.2	**1993**	83.0	**1983**	91.2	**1985**	82.4	**1985**	91.2	**2010**	73.6

**Table 2:** Top drought year based on drought severity (D1 to D4) estimated using domain mean drought indices for every month in the crop periods from 1981–2013.

Emergence					Heading				Anthesis			Maturity	
Year	Oct.	Year	Nov.	Year	Dec.	Year	Jan.	Year	Feb.	Year	Mar.	Year	Apr.
Meteorological Drought													
						**2007**	−1.1 (D1)	**2006**	−1.0 (D1)	**2004**	−0.8 (D1)		
Hydrological Drought													
**2000**	−1.6 (D3)	**1983**	−1.8 (D3)	**1998**	−1.9 (D4)	**1990**	−2.2 (D4)	**2006**	−2.2 (D4)	**2004**	−1.9 (D1)	**1990**	−1.5 (D2)
Soil Moisture Drought													
**1986**	−1.1 (D1)	**1989**	−1.1 (D1)	**1989**	−0.9 (D1)	**1990**	−1.0 (D1)	**2001**	−1.1 (D1)	**1985**	−0.8 (D1)	**1999**	−0.8 (D1)
Vegetation Drought													
**2000**	0.2 (D2)	**1993**	0.2 (D2)	**1993**	0.2 (D2)	**1983**	0.1 (D3)	**1985**	0.2 (D2)	**1985**	0.1 (D3)	**2010**	0.2 (D2)

**Table 3:** Time lag of the transformation from meteorological (Met.) to hydrological drought (Hyd.), meteorological to soil moisture drought (Soi.), hydrological to soil moisture drought, soil moisture to vegetation drought (Veg.), and meteorological to vegetation drought for 1981–2013.

Evolution	Met. – Hyd.	Met. – Soi.	Hyd. – Soi.	Soi. – Veg.	Met. – Veg.
Time lag	0	0	0	0	1

**Table 4:** Relationship between wheat yields with the domain drought indices for every month in the growing season.

Stage	Emergence		Heading		Anthesis		Maturity
Drought Type	Oct.	Nov.	Dec.	Jan.	Feb.	Mar.	Apr.
Meteorological	−0.01	0.02	−0.36*	−0.32	0.00	−0.22	0.31
Hydrological	0.24	0.22	−0.28	0.03	0.14	0.04	0.30
Soil Moisture	0.38*	0.45*	0.34	0.29	0.44*	0.06	0.30
Vegetation	0.55*	−0.08	−0.17	0.17	0.75*	0.74*	0.17

Numbers marked as * indicate passing the significant test (p < 0.05). From October to April, these p values are 0.03, 0.00, 0.01, 0.04, 0.01, 0.00, and 0.00. The sample of correlation analysis (n) was 33. Only the maximum, positive, and statistically significant correlation coefficients for every month are shown in bold text. Based on the Kolmogorov–Smirnov test (K–S test), the yield anomaly index was not normally distributed, therefore the Spearman correlation coefficient was used here.
