# Supplementary material for: Droughts in India from 1981 to 2013 and Implications to Wheat Production
Source: Sci Rep. 2017 Mar 15;7:44552. doi: 10.1038/srep44552 (PMC5353643; doi:10.1038/srep44552)
Supplement: Supplementary Information [file srep44552-s1.pdf]

[Scientific Reports]

Supporting Information for

Droughts in India from 1981 to 2013 and Implications to Wheat Production

Xiang Zhang[1,2], Renee Obringer[3], Chehan Wei[4], Nengcheng Chen[1,5,*], Dev Niyogi[2,3]

[1] State Key Laboratory of Information Engineering in Surveying, Mapping, and Remote Sensing (LIESMARS), Wuhan University, Wuhan 430079, China.

[2] Department of Agronomy-Crops, Soil, Environmental Science, Purdue University, West Lafayette, IN 47906, USA.

[3]Department of Earth, Atmospheric, and Planetary Sciences, Purdue University, West Lafayette, IN 47906, USA.

[4]Lyles School of Civil Engineering, Purdue University, West Lafayette, IN 47906, USA.

[5]Collaborative Innovation Center of Geospatial Technology, Wuhan 430079, China.

Corresponding author: Nengcheng Chen (cnc@whu.edu.cn)

Contents of this file

Text
Figures S1 to S7
Tables S1 to S10

Text

Drought determination by SPI/SRI/SSI. SPI[1], SRI[2], and SSI[3] are three standardized measurements for precipitation, runoff, and soil moisture deficit. For simplification and spatial comparability, SPI, SRI, and SSI are widely used to characterize droughts in India as well as world wide[4-7]. For example, SPI quantifies observed precipitation as a standardized departure from a selected probability distribution function that models the raw precipitation data. The raw precipitation data are typically fitted to a gamma distribution, and then transformed to a normal distribution. The SPI values can be interpreted as the number of standard deviations by which the observed anomaly deviates from the long-term mean. In the calculation of SRI and SSI, the standardization procedure has been rigorously tested by the Kolmogorov–Smirnov test, assuming normal, log–normal, poisson, exponential, rayleigh, and gamma distribution for runoff and soil moisture at the alpha level of 0.05. Total 62 grids were included in the test and the sample size for each grid is 396. It was found that about 73% runoff raw data was suitable to be represented by the gamma distribution, while none of grid was suitable to be represented by normal or log-normal distribution. Therefore, we adopted the gamma function to compute SRI. McKee at al.[1] suggested that the gamma distribution can also be applied to other variables relevant to drought, e.g., streamflow or reservoir contents. Shukla and Wood[2] also found gamma distribution may perform better for low runoff values. In terms of the SSI, the current practice is to adopt a normal[8] or non-parametric empirical distribution approach[9]. However, no soil moisture grid was suitable to be represented by any of the above functions in this study area. This result is probably likely due to the extensive soil moisture management in the study area (i.e., irrigation). Due to the highly correlated relationship with runoff, we also adopted the gamma distribution here in calculating SSI. However, we believe additional research is needed to find a more appropriate distribution to fit the soil moisture values in this intensively irrigated area.

Index values with corresponding severities are shown in Supplementary Table S9. Categories D1-D4 were judged as drought events. These thresholds were adopted from the United States Drought Monitor (USDM) described in Svoboda et al.[10]. This drought category uses a percentile approach to classify the severity, as shown in the Table S9. This approach also enables the user to easily interpret the probability of one drought event in terms of the number of events per 100 years. For example, D0 (abnormally dry) conditions indicate a 21% to 31% chance of occurring in any given year at a given location, while D1 (moderate drought) events occur 11% to 20% of the time[10]. It is noted that this classification system is slightly different from the World Meteorological Organization (WMO) recommended system by McKee et al.[1]. Both systems use the probability of occurrence to determine drought severity. But the adopted system can provide five finer drought categories, compared with the three categories in McKee et al.[1] In addition, the threshold of 0.35 in VCI also accords with the original study by Kogan[11].

Computation of VCI. VCI is a pixel-wise normalization of NDVI that is useful for making relative assessment of changes in the NDVI signal by filtering out the contribution of local geographic features to the spatial variability of NDVI[11]. The VCI is computed as equation (1).

$$VCI_i = \frac{NDVI_i - NDVI_{\min}}{NDVI_{\max} - NDVI_{\min}} \quad (1)$$

where $NDVI_i$ is the smoothed weekly NDVI at each pixel, and $NDVI_{\max}$ and $NDVI_{\min}$ are the absolute maximum and minimum NDVI of each pixel, respectively. The VCI smoothes out non-uniformity in the AVHRR data and it is an indicator of how weather conditions have influenced the relative vigor of the vegetation with respect to the ecologically defined limits[12]. The VCI has been widely evaluated and applied, and was found to be suitable for agricultural drought[13-15].

The sensitivity analysis used here is similar to that of Anderson et al.[16]: the absolute sensitivity (S_V) of any of the output variable (VCI) to ±X uncertainty in NDVI was assigned as equation (2).

$$S_V = \left|(V_{X+} - V_{X-}) / V_{xr}\right| \quad (2)$$

Where V_{X+} and V_{X-} are the estimated VCI variables when the value NDVI are increased or decreased by X, and V_{xr} is the value of the estimated VCI variable at actual NDVI. Based on this sensitivity analysis, it was found the uncertainty of NDVI has no impact on the VCI value. Therefore, VCI was relatively immune to the uncertainty of absolute NDVI values.

Drought Evolution Mechanism. Generally speaking, the meteorological drought is often the first kind of drought to occur. A deficit of precipitation during a certain period of time leads to the shortage of water on the land surface. Along with the high temperature and wind, potential evapotranspiration increases to consume more water. When the water balance in the soil disrupts, water on the surface or subsurface (i.e., streamflow, reservoir, and groundwater) can be transferred into the soil by irrigation system. Therefore, although the soil moisture deficit occurred earlier than hydrological water deficit from the theoretical perspective[17,18], their occurrence order is usually reversed in irrigation agriculture. This study selected one of the main wheat production regions in India. The irrigation in this area is pervasive after the Green Revolution took place in the 1960s. Therefore, in this study, the soil moisture drought is believed to occur after hydrological drought. After the soil moisture drought, vegetation is under water-stress. Though it has limited adaptive functions to decrease the water consumption (i.e., stoma closure), plant can have permanent damages after a period of water-stress wilting, which can then result in yield loss. This is the final drought to occur: a vegetation drought. The above analysis is the theoretical support of this study to

investigate these four kinds of droughts at the same time, which is also shown in Supplementary Fig. S7. We acknowledge that the above drought evolution sequence and time interval are varied in different locations. This also demonstrates the necessity to conduct a comprehensive and local-scale drought study as a system to gain knowledge to support drought mitigation.

Cross-correlation for drought evolution. Cross-correlation (or lagged correlation) refers to the correlation between two time series shifted in time relative to one another. For two time series, data of $\{x_1, x_2, ..., x_n\}$ and $\{y_1, y_2, ..., y_n\}$, the cross-correlation coefficient $r_{xy}(k)$ at lag *k* is estimated by:

$$r_{xy}(k) = \frac{C_{xy}(k)}{S_x S_y} \tag{3}$$

$$C_{xy}(k) = \begin{cases} \frac{1}{n}\sum_{t=1}^{n-k}(x_t - \bar{x})(y_{t+k} - \bar{y}), k = 0,1,2,... \\ \frac{1}{n}\sum_{t=1}^{n+k}(y_t - \bar{y})(x_{t-k} - \bar{x}), k = -1,-2,... \end{cases} \tag{4}$$

$$S_x = \sqrt{\frac{1}{n}\sum_{t=1}^{n}(x_t - \bar{x})^2} \qquad S_y = \sqrt{\frac{1}{n}\sum_{t=1}^{n}(y_t - \bar{y})^2} \tag{5}$$

It is obvious that if the time lag k is equal to 0, the cross-correlation became the commonly used Pearson correlation. When the value of k changes, the correlation coefficient $r_{xy}(k)$ changes accordingly. When the correlation coefficient reached the maximum value, the time lag k is regarded as the statistical time lag that existed between two variables over time. Shorter time lags indicate faster drought evolution processes between two kinds of drought, while longer time lags represent long-term evolution processes. When responding to drought, it is often useful to know how fast the meteorological drought will evolve into a hydrological or vegetation drought. The sample of correlation uses the total 396 monthly drought data.

Mann-Kendall test with Sen's slope for drought trend analysis. The Mann-Kendall test is based on the correlation between the ranks of a time series and their time order. For a time series $X = \{x_1, x_2, ..., x_n\}$, the test statistic *S* is given by

$$S = \sum_{i=1}^{n-1}\sum_{j=i+1}^{n} a_{ij} \tag{6}$$

where

$$a_{ij} = sign(x_j - x_i) = sign(R_j - R_i) = \begin{cases} 1, x_i < x_j \\ 0, x_i = x_j \\ -1, x_i > x_j \end{cases} \quad (7)$$

where R_i and R_j are the ranks of observations x_i and x_j of the time series, respectively. As can be seen from equation (6), the test statistic depends only on the rank of the observations, rather than their actual values, resulting in a distribution-free test statistic. Therefore, the Mann–Kendall trend test is not affected by the actual distribution of the data and is less sensitive to outliers. On the other hand, parametric trend tests, although more powerful, require the data to be normally distributed and are more sensitive to outliers. Therefore, the Mann–Kendall test, as well as other non-parametric trend tests, prove more suitable for detecting trends in a hydrological time series which are usually skewed and may be contaminated with outliers.

Under the assumption that the data are independent and identically distributed, the mean and variance of the S statistic in equation (6) are given by[19]

$$E(S) = 0 \quad (8)$$

$$V_0(S) = n(n-1)(2n+5)/18 \quad (9)$$

where *n* is the number of observations. The existence of tied ranks (equal observations) in the data results in a reduction of the variance of *S* to become

$$V_0^*(S) = n(n-1)(2n+5)/18 - \sum_{j=1}^{m} t_j(t_j - 1)(2t_j + 5)/18 \quad (10)$$

where *m* is the number of groups of tied ranks, each with t_j tied observations.

Kendall[19] also showed that the distribution of *S* tended to normality as the number of observations becomes larger. The significance of trends can be tested by comparing the standardized variable *Zs* in equation (11) with the standard normal variate at the desired significance level α, where the subtraction or addition of unity in equation (11) is a continuity correction.

$$Z_s = \begin{cases} (S-1)/\sqrt{V_0^*(S)}, S > 0 \\ 0 \\ (S+1)/\sqrt{V_0^*(S)}, S < 0 \end{cases} \quad (11)$$

Positive values of *Zs* indicate increasing trends while negative *Zs* values show decreasing trends. In this study, trends were estimated on the different drought indices (SPI, SRI, SSI, and VCI) to identify statistically significant changes in different drought forms. If a significant trend is found, the rate of change can further be calculated using the Sen's slope estimator[20]. The Sen's method uses a linear model to estimate the slope of the trend, and the variance of the residuals should be constant in time calculated as:

$$Q_i = \frac{X_j - X_k}{j - K}, i = 1, ..., n \quad (12)$$

where X_j and X_k are data values at times j and k (j >k), respectively. If there is only one datum in each time period, then N=n(n-1)/2, where n is the number of time periods. The n values of Qi are ranked from smallest to largest, and the median of slope or Sen's slope estimator is computed as:

$$Q_{med} = \begin{cases} Q_{[(n+1)/2]}, \ if \ n \ is \ odd \\ \frac{Q_{[n/2]} + Q_{[(n+2)/2]}}{2}, \ if \ n \ is \ even \end{cases} \quad (13)$$

The Q_{med} sign reflects data trend, while its value indicates the steepness of the trend. This estimator can be computed efficiently, and is insensitive to outliers. It can be significantly more accurate than non-robust simple linear regression for skewed and heteroskedastic data, and competes well against non-robust least squares even for normally distributed data in terms of statistical power.

Yield Anomalies Index (YAI) calculation. The Yield Anomalies Index (YAI) for every year was calculated using the following formula:

$$YAI = (Y - \mu) / \sigma \quad (14)$$

where Y is the crop yield in one certain year, μ is the average yield during a long term, and σ is the standard deviation of long-term yield.

Figure S1. Mean (a) SPI-1, (b) SRI-1, (c) SSI-1, and (d) VCI in the study area for every month from 1981 to 2013 to determine meteorological, hydrological, soil moisture, and vegetation drought, respectively.

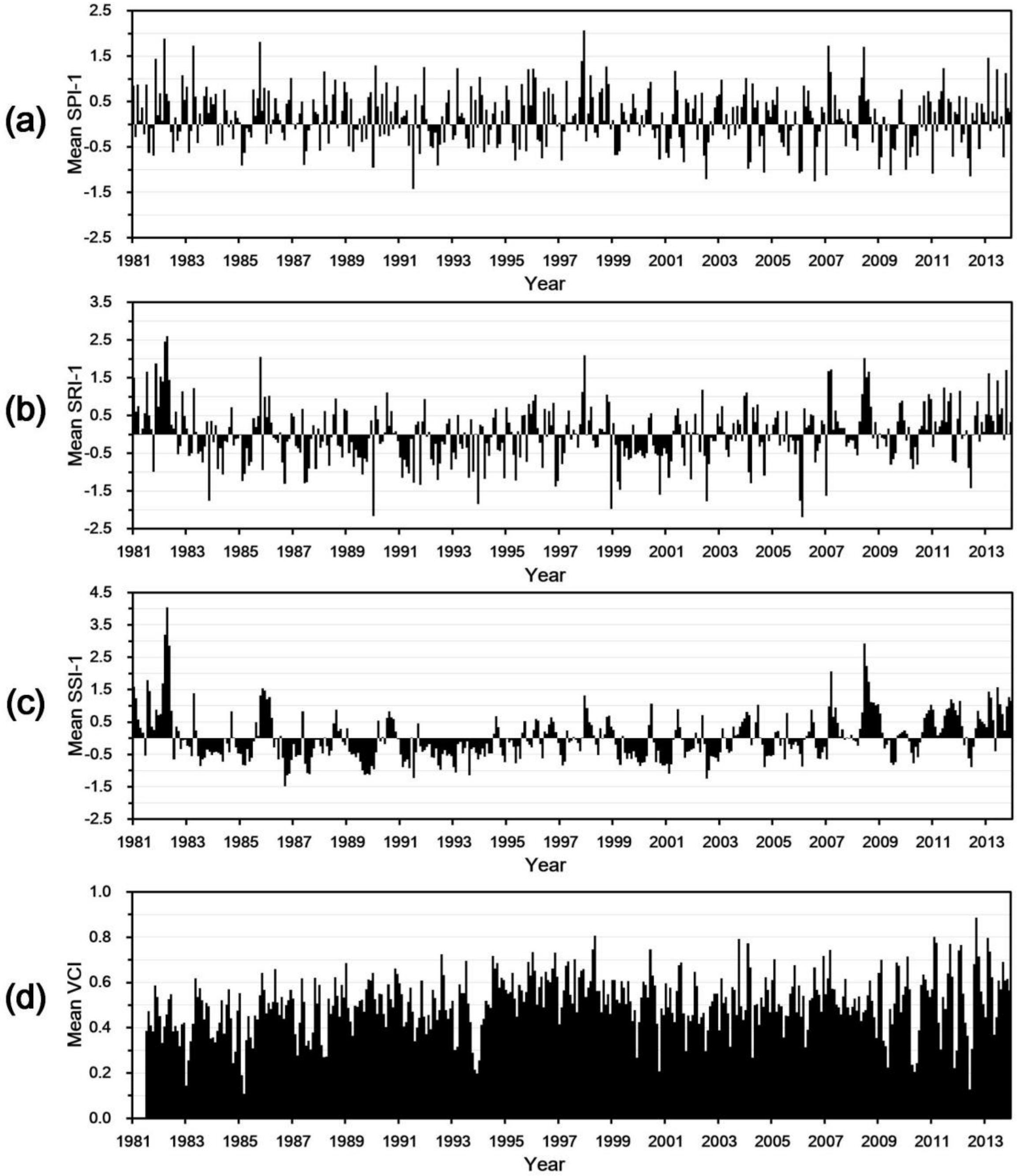

Figure S2. Linear regression of mean areal extent percentage of (a) meteorological drought, (b) hydrological drought, (c) soil moisture drought, and (d) vegetation drought from 1981-2013.

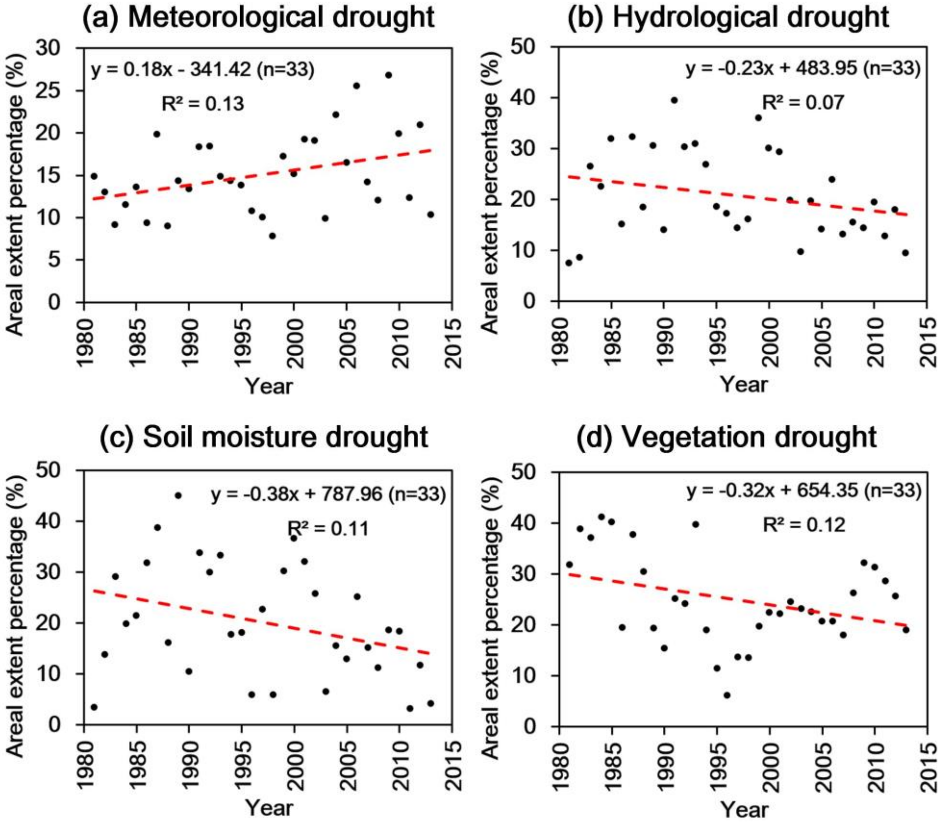

Figure S3. Latitudinal variations for the temporal extent of droughts in each triennium for the seven wheat growth months during 1981–2013. For the middle panel, x and y axis values are the same for the left and right panels. The latitudinal variations data was calculated and color rendered by Matlab R2014b (Version 8.4, URL: http://www.mathworks.com) [Software] with the method described in the next section. Finally all these maps were organized and labeled in the Microsoft Visio Professional 2013 (Version 15.0.4569.1506, URL: https://products.office.com/en-us/visio) [Software].

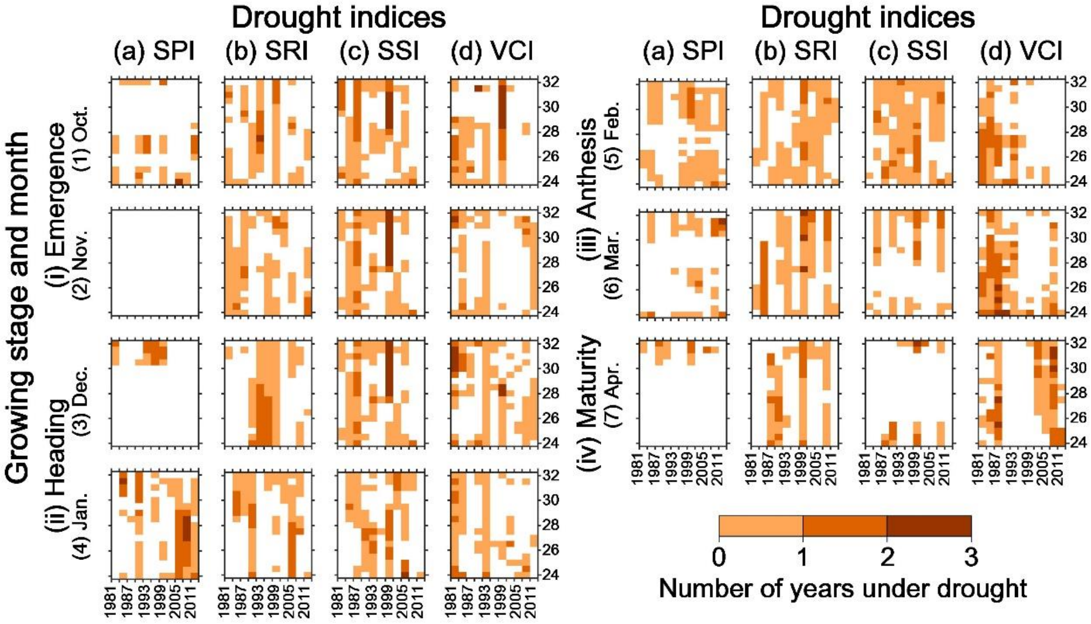

Figure S4. Scattering plot between YAI and the final PADI value for September 2012 in twelve states of the Midwest. Based on the Kolmogorov–Smirnov test (K–S test) at an alpha level of 0.05, YAI is not normally distributed. Therefore, Spearman's rank correlation value (r) and linear regression line are given. Correlation coefficient (r) with spark (*) indicates $p < 0.05$ in the significance test. From (a) to (f), these p values are 0.03, 0.00, 0.01, 0.01, 0.00, and 0.00.

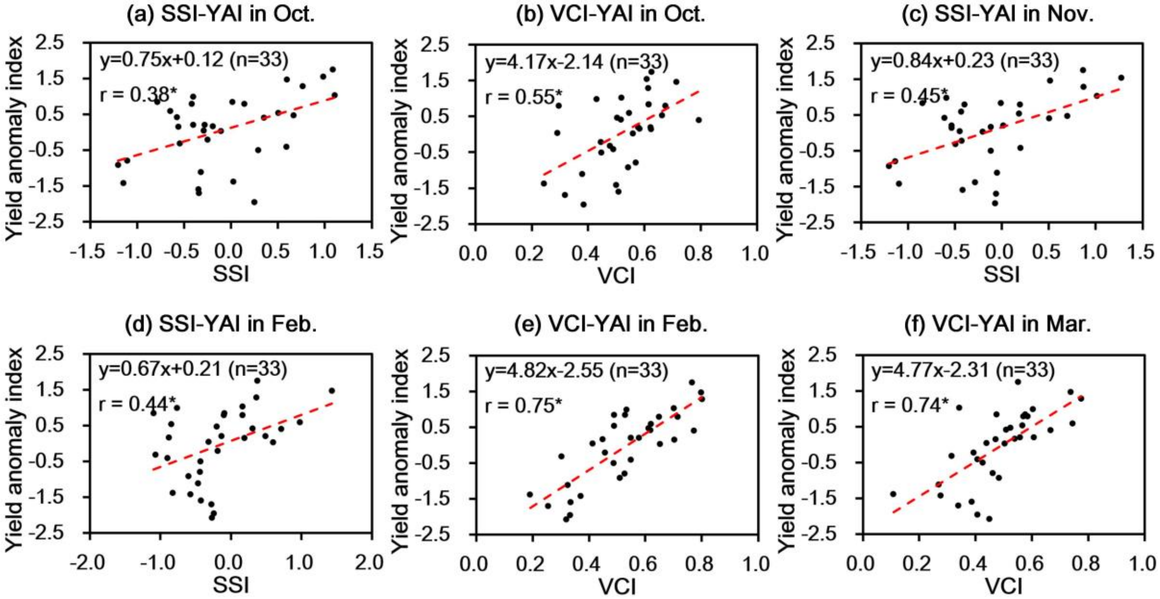

Figure S5. Study area with monthly mean precipitation, temperature, land cover, and relative location in India. It was generated by ArcGIS Desktop (Version 10.2.3348, URL: http://www.esri.com) [Software]. Two map layers were used in this figure, including administrative boundary layer and land cover layer. Administrative boundary and land cover data were obtained from DIVA-GIS (URL: http://www.diva-gis.org/Data). DIVA-GIS provides free spatial data for geographical information system. Precipitation and temperature data were retrieved from Yr, which is a joint service by the Norwegian Meteorological Institute and the Norwegian Broadcasting Corporation (URL: https://www.yr.no/place/India/). These data and products are licensed under Norwegian license for public data (NLOD; http://data.norge.no/nlod/en/1.0) and Creative Commons Attribution 3.0 Norway (https://creativecommons.org/licenses/by/3.0/no/), and they are freely available to the public for use, distribution and processing.

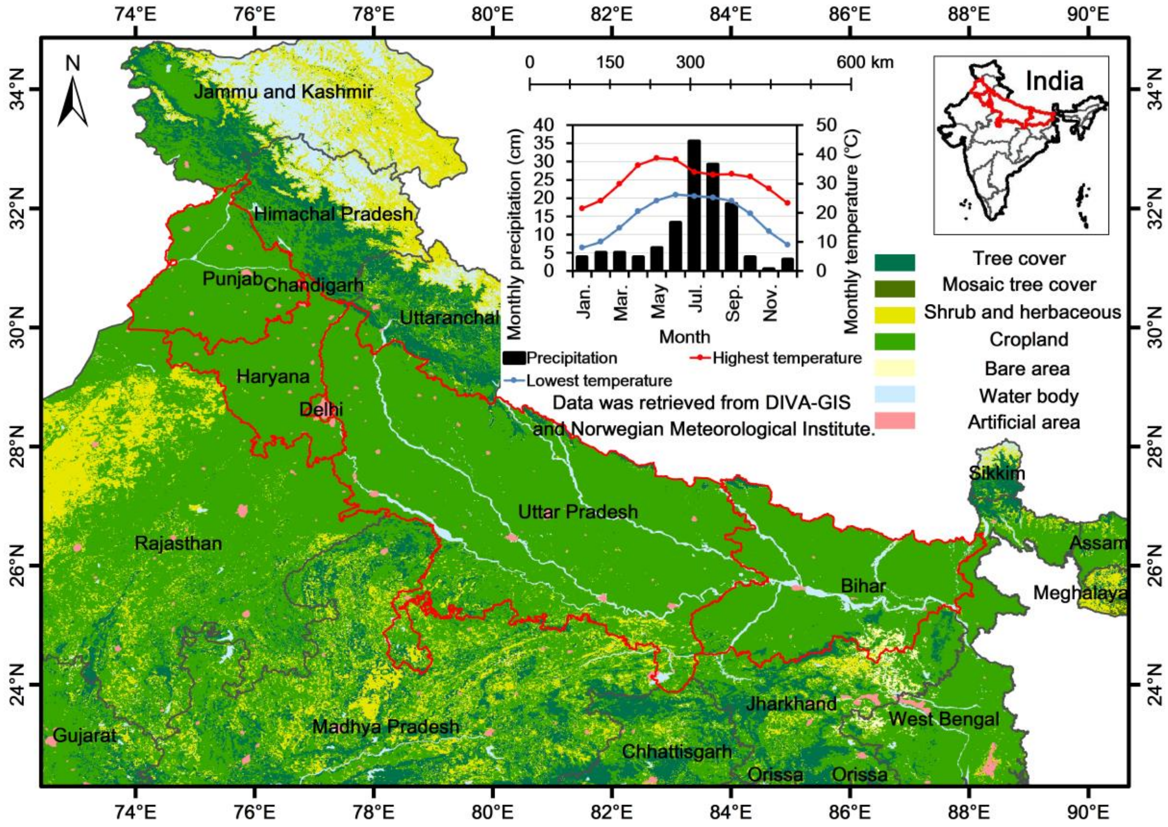

Figure S6. Total wheat production, area, and mean yield in the study area from 1980 to 2014. The red line is the linear fitting trend of wheat yield.

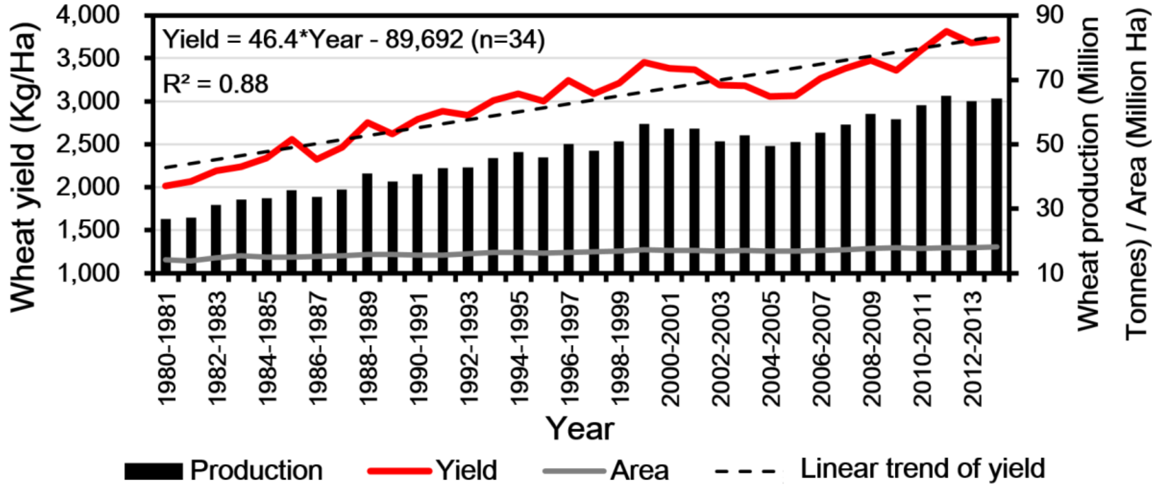

Figure S7. Flowchart of drought evolution mechanism from meteorological, to hydrological, to soil moisture, and to vegetation drought.

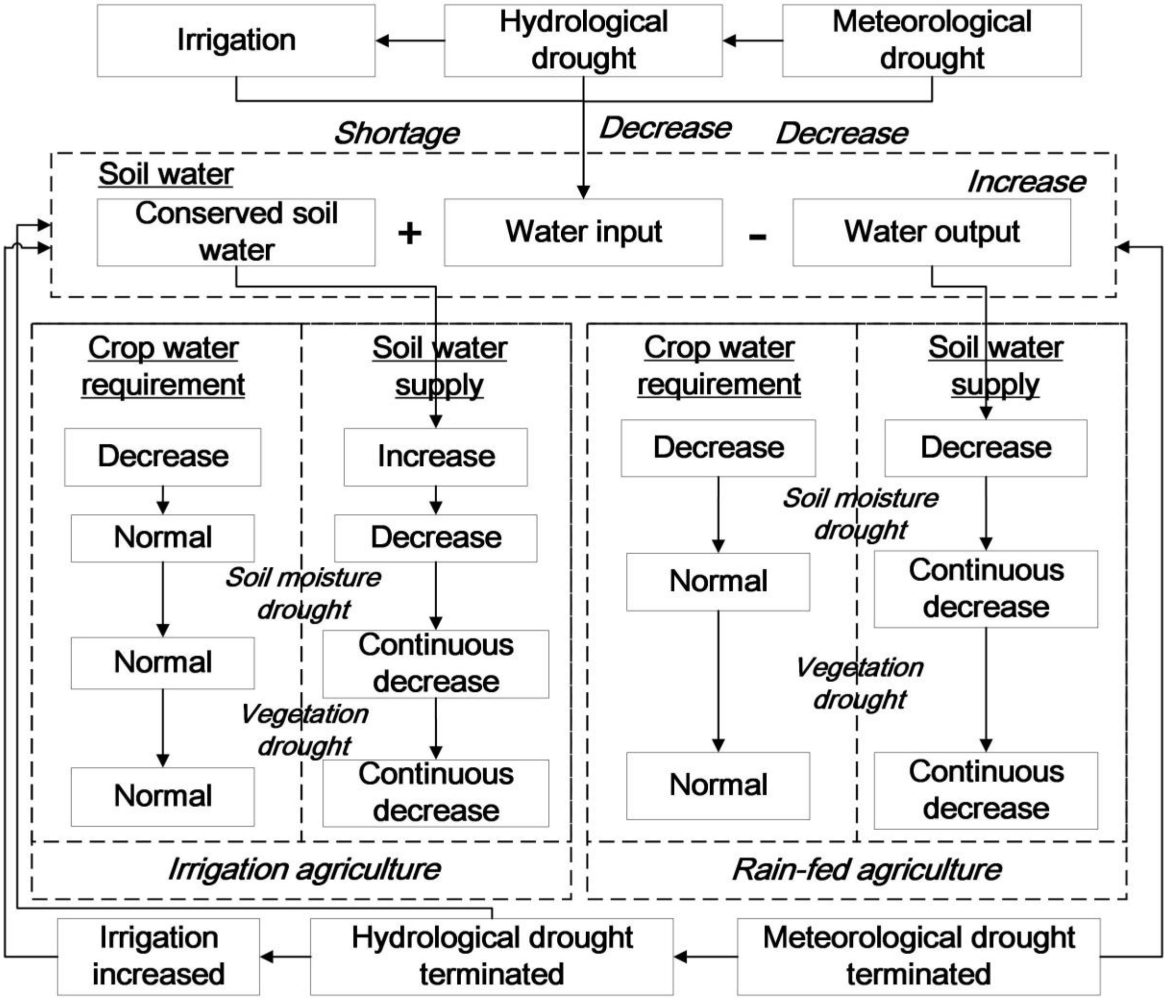

Table S1. Occurrence of meteorological droughts with moderate or higher severities estimated using domain-averaged drought indices for every month in the crop periods. Years when at least three types of droughts occurred are marked in bold.

Stage	Emergence		Heading		Anthesis		Maturity
Year	October	November	December	January	February	March	April
**1985**					D1		
**1990**				D1			
**1997**					D1		
1998							
**2004**					D1	D1	
**2006**				D1	D1		
2007				D1			
2009				D1			
**2010**				D1			
2011				D1			

Table S2. Occurrence of hydrological droughts with moderate or higher severities estimated using domain-averaged drought indices for every month in the crop periods. Years when at least three types of droughts occurred are marked in bold.

Stage	Emergence		Heading		Anthesis		Maturity
Year	October	November	December	January	February	March	April
1981	D1						
1983		D3					
1984						D1	
**1985**		D1			D1	D1	
1987		D1					
1989							D1
**1990**				D4			
1991	D2				D1		D1
1992			D1				D1
**1993**	D1		D3				
1994			D1			D1	
1996		D2	D1				
**1997**							
1998			D3				
**1999**						D1	D2
**2000**	D3						
**2001**			D1		D1		
**2004**					D1	D1	
**2006**				D3	D4		
2007				D3			
**2010**							D1

Table S3. Occurrence of soil moisture droughts with moderate or higher severities estimated using domain-averaged drought indices for every month in the crop periods. Years when at least three types of droughts occurred are marked in bold.

Stage	Emergence		Heading		Anthesis		Maturity
Year	October	November	December	January	February	March	April
**1985**					D1	D1	
1986	D1	D1					
1989	D1	D1	D1				
**1990**				D1			
1991					D1		
1992							
**1993**				D1	D1		
**1997**					D1		
**1999**							D1
**2000**		D1	D1	D1			
**2001**					D1	D1	
**2006**					D1		

Table S4. Occurrence of vegetation droughts with moderate or higher severities estimated using domain-averaged drought indices for every month in the crop periods. Years when at least three types of droughts occurred are marked in bold.

Stage	Emergence		Heading		Anthesis		Maturity
Year	October	November	December	January	February	March	April
1982	D1				D1		
1983			D1	D3	D1	D1	
1984	D2	D1			D1		
**1985**					D2	D3	D1
1987						D1	
1988					D1	D1	D1
**1993**	D1	D2	D2		D1	D1	
1994				D2			
**1999**			D1				
**2000**	D2						
**2001**	D1						
**2004**							D1
**2006**							D1
2009						D1	D1
**2010**							D2
2011		D2	D1				

Table S5. Concurrent meteorological, hydrological, soil moisture, and vegetation droughts with moderate or higher severities estimated using domain-averaged drought indices for every month in the crop periods. They are represented by M, H, S, and V, respectively. The symbol of “+” represents the concurrent situation.

Stage	Emergence		Heading		Anthesis		Maturity
Year	October	November	December	January	February	March	April
1985					M+H+S+V	H+S+V	
1990				M+H+S			
1991					H+S		
1993	H+V		H+V		S+V		
1997					M+S		
1999							H+S
2000	H+V						
2001					H+S		
2004					M+H	M+H	
2006				M+H	M+H+S		
2007				M+H			
2010							H+V

Table S6. Mean duration of meteorological, hydrological, soil moisture, and vegetation drought time determined by domain-averaged SPI, SRI, SSI, and VCI during 1981-1989, 1990-1999, and 2000-2013, respectively. Ave. is short for average duration. Ran. is short for duration range. Std. is short for standard deviation. Sample size of each decade is 9, 10, and 10, respectively. Sample size (n) for meteorological drought in each decade is 2, 5, and 6. Sample size (n) for hydrological drought in each decade is 8, 9, and 7. Sample size (n) for soil moisture drought in each decade is 5, 5, and 6. Sample size (n) for vegetation drought in each decade is 6, 3, and 7. Units are in months.

Decade	Meteorological Drought			Hydrological Drought			Soil Moisture Drought			Vegetation Drought		
	Ave.	Ran.	Std.	Ave.	Ran.	Std.	Ave.	Ran.	Std.	Ave.	Ran.	Std.
1981-1989	**1**	1-1	0	**1.2**	1-2	0.4	**2.8**	1-6	1.9	**1.9**	1-3	0.7
1990-1999	**1**	1-1	0	**1.2**	1-2	0.4	**1.3**	1-2	0.4	**1.7**	1-3	1.2
2000-2009	**1.2**	1-1.5	0.3	**1.1**	1-1.5	0.2	**1.4**	1-2	0.5	**1.3**	1-3	0.8

Table S7. Frequency of meteorological, hydrological, soil moisture, and vegetation drought time determined by domain-averaged SPI, SRI, SSI, and VCI during 1981-1989, 1990-1999, and 2000-2013, respectively. Units are the number of droughts per decade.

Decades	Meteorological Drought	Hydrological Drought	Soil Moisture Drought	Vegetation Drought
1981-1989	2	13	5	20
1990-1999	5	21	10	8
2000-2009	11	11	10	9

Table S8. Mean areal extent of meteorological, hydrological, soil moisture, and vegetation drought time determined by pixel level of SPI, SRI, SSI, and VCI during 1981-1989, 1990-1999, and 2000-2013, respectively. Ave. is short for average areal extent. Ran. is short for areal extent range. Std. is short for standard deviation. Sample size (n) in each decade is 9, 10, and 10, respectively. Unit is percentage (%).

Decades	Meteorological Drought			Hydrological Drought			Soil Moisture Drought			Vegetation Drought		
	Ave.	Ran.	Std.	Ave.	Ran.	Std.	Ave.	Ran.	Std.	Ave.	Ran.	Std.
1981-1989	**12.7**	9.0-19.8	3.5	**21.4**	7.4-32.2	9.7	**24.3**	3.4-45.0	13.0	**32.9**	19.3-41.2	8.5
1990-1999	**13.9**	7.8-18.4	3.6	**24.3**	14.0-39.5	9.5	**20.7**	5.8-33.8	10.9	**18.7**	6.1-39.7	9.4
2000-2009	**18.0**	9.9-26.8	5.6	**18.9**	9.6-30.0	6.9	**19.9**	6.5-36.6	9.7	**23.2**	17.9-32.1	3.8

Table S9. Ranges of drought indices (SPI, SRI, SSI, and VCI) for various drought severities and categories as described in Svoboda et al.[10].

Drought Severity	SPI, SRI, and SSI	VCI	Category	Percentile Chance
Abnormally dry	-0.50 to -0.79	0.45 to 0.36	D0	20 to 30
Moderate drought	-0.80 to -1.29	0.26 to 0.35	D1	10 to 20
Severe drought	-1.30 to -1.59	0.25 to 0.16	D2	5 to 10
Extreme drought	-1.60 to -1.99	0.15 to 0.06	D3	2 to 5
Exceptional drought	-2.00 or less	0.00 to 0.05	D4	0 to 2

Table S10. Phenological stages for winter wheat crops (source: Steduto et al.[21]; Water Development and Management Unit[22]).

Stage	Description	Date	Yield response factor
Emergence	Germination to emergence	October to November	0.2
Heading	From emergence to double ridge	December to January	0.6
Anthesis	From double ridge to anthesis	February to March	0.5
Maturity	Includes the grainfilling period, from anthesis to maturity	April	-

Reference

1. McKee, T. B., Doesken, N. J. & Kleist, J. *The relationship of drought frequency and duration to time scales in Proceedings of the 8th Conference on Applied Climatology.* 179-183 (American Meteorological Society Boston, MA, 1993).
2. Shukla, S. & Wood, A. W. Use of a standardized runoff index for characterizing hydrologic drought. *Geophys. Res. Lett.* **35**, 1-7, doi:10.1029/2007GL032487 (2008).
3. Hao, Z. & AghaKouchak, A. Multivariate standardized drought index: a parametric multi-index model. *Adv. Water Resour.* **57**, 12-18, doi:10.1016/j.advwatres.2013.03.009 (2013).
4. Bhuiyan, C., Singh, R. P. & Kogan, F. N. Monitoring drought dynamics in the Aravalli region (India) using different indices based on ground and remote sensing data. *Int. J. Appl. Earth Obs. Geoinf.* **8**, 289-302, doi:10.1016/j.jag.2006.03.002 (2006).
5. Thomas, T., Nayak, P. C. & Ghosh, N. C. Spatiotemporal analysis of drought characteristics in the bundelkhand region of central India using the standardized precipitation index. *J. Hydrol. Eng.* **20**, doi:10.1061/(asce)he.1943-5584.0001189 (2015).
6. Yadav, R. R., Misra, K. G., Yadava, A. K., Kotlia, B. S. & Misra, S. Tree-ring footprints of drought variability in last similar to 300 years over Kumauni Himalaya, India and its relationship with crop productivity. *Quat. Sci. Rev.* **117**, 113-123, doi:10.1016/j.quascirev.2015.04.003 (2015).
7. AghaKouchak, A. *et al.* Remote sensing of drought: progress, challenges and opportunities. *Rev. Geophys.* **53**, 452-480, doi:10.1002/2014rg000456 (2015).
8. Mishra, V. & Cherkauer, K. A. Retrospective droughts in the crop growing season: Implications to corn and soybean yield in the Midwestern United States. *Agric. For. Meteorol.* **150**, 1030-1045, doi: 10.1016/j.agrformet.2010.04.002 (2010).
9. Hao, Z. & AghaKouchak, A. Multivariate standardized drought index: a parametric multi-index model. *Adv. Water. Resour.* **57**, 12-18, doi:10.1016/j.advwatres.2013.03.009 (2013).
10. Svoboda, M. *et al.* The drought monitor. *Bull. Am. Meteorol. Soc.* **83**, 1181-1190, doi:10.1175/1520-0477(2002)083<1181:TDM>2.3.CO;2 (2002).
11. Kogan, F. N. Application of vegetation index and brightness temperature for drought detection, *Adv. Space Res.* **15**, 91-100, doi:10.1016/0273-1177(95)00079-T (1995).
12. Unganai, L. S. & Kogan F. N. Drought monitoring and corn yield estimation in Southern Africa from AVHRR data, *Remote Sens. Environ.* **63**, 219-232, doi: 10.1016/S0034-4257(97)00132-6 (1998).
13. Singh, R. P., Roy, S. & Kogan, F. Vegetation and temperature condition indices from NOAA AVHRR data for drought monitoring over India. *Int. J. Remote Sens.* **24**, 4393-4402, doi:10.1080/0143116031000084323 (2003).
14. Jain, S. K., Keshri, R., Goswami, A., Sarkar, A. & Chaudhry, A. identification of drought-vulnerable areas using NOAA AVHRR data. *Int. J. Remote Sens.* **30**, 2653-2668, doi:10.1080/01431160802555788 (2009).

15. Quiring, S. M. & Ganesh, S. Evaluating the utility of the vegetation condition index (VCI) for monitoring meteorological drought in Texas. *Agric. For. Meteorol.* **150**, 330-339, doi:10.1016/j.agrformet.2009.11.015 (2010).
16. Anderson, M. C., Norman, J. M., Diak, G. R., Kustas, W. P. & Mecikalski, J. R. A two-source time-integrated model for estimating surface fluxes using thermal infrared remote sensing. *Remote Sens. Environ.* **60**, 195-216, doi: 10.1016/S0034-4257(96)00215-5 (1997).
17. National Weather Service, What is Drought Why is Drought Important. http://www.nws.noaa.gov/os/brochures/climate/Drought2.pdf (2008) (Data of access: 21/10/2016)
18. National Drought Mitigation Center, Types of Drought. http://drought.unl.edu/DroughtBasics/TypesofDrought.aspx (2016) (Data of access: 21/10/2016)
19. Kendall, M. G. *Rank Correlation Methods.* 1-272 (Oxford University Press, 1948).
20. Sen, P. K. Estimates of the regression coefficient based on Kendall's tau. *J. Am. Stat. Assoc.* **63**, 1379-1389 (1968).
21. Steduto, P., Hsiao, T. C., Fereres, E. & Raes, D. Crop yield response to water. http://www.fao.org/docrep/016/i2800e/i2800e00.htm (2012) (Data of access: 21/10/2016).
22. Water Development and Management Unit. Crop Water Information: Wheat. http://www.fao.org/nr/water/cropinfo_wheat.html (2015) (Data of access: 21/10/2016)
